# The Brazilian version of Skindex-16 is a valid and reliable instrument to assess the health-related quality of life of patients with skin diseases

**DOI:** 10.1371/journal.pone.0194492

**Published:** 2018-03-22

**Authors:** Cristiane Botelho Miranda Cárcano, Cleyton Zanardo de Oliveira, Bianca Sakamoto Ribeiro Paiva, Carlos Eduardo Paiva

**Affiliations:** 1 Department of Prevention, Barretos Cancer Hospital, Barretos, Brazil; 2 Dr. Paulo Prata School of Health Sciences (Faculdade de Ciências da Saúde Dr. Paulo Prata – FACISB), Barretos, Brazil; 3 Researcher Support Center, Barretos Cancer Hospital, Barretos, Brazil; 4 Palliative Care and Health-Related Quality of Life Research Group, Barretos, Brazil; 5 Department of Clinical Oncology, Barretos Cancer Hospital, Barretos, Brazil; Ohio State University Wexner Medical Center, UNITED STATES

## Abstract

**Introduction:**

The aim of this study was to assess the psychometric properties of the Brazilian version of Skindex-16 in patients with various skin diseases.

**Methods:**

Dermatologic assessments were performed for the diagnosis and classification of the severity of skin conditions. The clinical feasibility of Skindex-16 was assessed based on the time required to complete the questionnaire and the number of unanswered items. The participants (n = 110) answered the Hospital Anxiety and Depression Scale (HADS), the Dermatology Life Quality Index (DLQI) and the Skindex-16 (Portuguese/Brazil version) questionnaires. Convergent validity was assessed based on the correlation of the Skindex-16 with the DLQI and HADS subscales. Known-groups validity was assessed based on the comparison of the mild, moderate and severe disease groups using the Kruskal-Wallis test. Internal consistency was assessed using Cronbach’s alpha and test-retest reproducibility using the intraclass correlation coefficient (ICC) obtained with 29 participants who answered the Skindex-16 a second time 3 to 10 days after the first assessment.

**Results:**

The mean time to answer the questionnaire was 2 min 41 sec. Cronbach’s alpha scores were 0.867, 0.930 and 0.888 for the Skindex-16 domains symptoms, emotions and functioning, respectively. The ICCs were 0.947, 0.860 and 0.843 for the Skindex-16 domains symptoms, emotions and functioning, respectively. All three Skindex-16 scales exhibited strong correlations with DLQI. Moderate correlations were found between HADS subscales and the Skindex-16 emotions domain. Known-groups validity showed differences in all three Skindex-16 domains between the mild and moderate skin disease groups (emotions: p < 0.001; symptoms: p = 0.049; functioning: p < 0.001) and between the mild and severe skin disease groups (emotions: p = 0.002; symptoms: p = 0.001; functioning: p = 0.002).

**Conclusion:**

The Portuguese/Brazil version of Skindex-16 is a valid and reliable instrument to assess the quality of life of patients with skin diseases.

## Introduction

Most skin diseases do not pose a direct risk to life, but the frequent compromises in physical appearance and emotional state can negatively affect the health-related quality of life (HR-QOL) [1]. A study that estimated the global burden of disease attributable to skin diseases from 1990 to 2010 for 187 countries found that collectively, these conditions were the fourth-leading cause of nonfatal disease burden expressed as years lost due to disability [2]. Several skin diseases might cause disability and handicap [3].

The relevance of HR-QOL in dermatology is demonstrated by the large number of recent studies on this subject. HR-QOL is one of the most important outcomes in dermatology as skin diseases are usually chronic and have a strong impact of skin diseases on social relationships, emotional state, work performances and daily activities [4]. The impact of disease on HR-QOL is increasingly emphasized as a relevant outcome in therapeutic clinical trials [5].

HR-QOL instruments have been used in clinical trials targeting a large number of skin diseases, especially chronic and recurrent conditions, such as acne, psoriasis, atopic dermatitis and contact dermatitis [4, 6–9].

As patient satisfaction and comfort has become increasingly important within the doctor-patient relationship, doctors are increasingly interested in instruments for objective assessments of HR-QOL [4]. Skindex-16 is an instrument that specifically assesses the effects of skin disorders on QOL, regardless of the type of dermatosis or patient’s comorbidity. Derived from Skindex-29, the questionnaire consists of 16 items and is easy to apply [10, 11]. A translated Brazilian Portuguese version of Skindex-16 is already available. In the present study, we assessed the psychometric properties of the Brazilian version of Skindex-16 on a sample of patients with different skin conditions.

## Methods

### Ethics issues

The present study complied with the ethics standards formulated in the Brazilian National Health Council Resolution 466/2012. The study was approved by the research ethics committee of Barretos Cancer Hospital (BCH, Barretos, São Paulo, Brazil, BCH no. 908/2015). All participants voluntarily signed an informed consent form.

### Study design and setting

The present methodological study sought to validate an instrument for health assessment. The Mapi Research Institute gave authorization for the validation of the Portuguese/Brazil version of Skindex-16. The data were collected at BCH, a large-size healthcare institution exclusively devoted to the treatment of cancer patients, and São Sebastião Foundation (SSF), a healthcare facility with an outpatient clinic for patients with skin disorders. Both BCH and SSF are located in the city of Barretos (São Paulo, Brazil).

This manuscript follows the Strengthening the Reporting of Observational Studies in Epidemiology (STROBE) statement on observational studies (S1 File).

### Case series

Patients aged 18 years old or older, able to communicate in the Brazilian Portuguese language, treated at SSF and BHC for any skin disease were included in the study. Patients with neuropsychiatric disorders who were unable to understand and answer the questionnaires were excluded from the study, as were patients who refused to participate in the study or to sign the informed consent form.

A total of 110 outpatients were included in the study from February 2015 to June 2016. The participants were selected by convenience sampling.

### Data collection

Data collection targeted sociodemographic and clinical information, including age, gender, marital status and educational level.

All participants were examined by the same dermatologist. Participants were asked to name their main skin complaint (defined as skin condition 1) and others, if present, which were considered secondary. Following diagnosis, the skin disorders were classified according to their severity or intensity as mild, moderate or severe. Patients were categorized under inflammatory diseases (such as psoriasis, eczematous dermatitis, or leprosy) and localized skin lesions (such as benign growths or skin cancer) [10].

All participants answered three instruments for health assessment: the Hospital Anxiety and Depression Scale (HADS), the Dermatology Life Quality Index (DLQI) and the Skindex-16 questionnaire. The participants could choose whether they preferred to answer the instruments by themselves or have them applied by an interviewer. The time needed to answer the Skindex-16 was measured. Items unanswered due to doubts or lack of understanding were identified and recorded by the interviewer.

To assess test-retest reliability, 29 participants were reassessed by the same investigator 3 to 10 days after the first assessment. On that occasion, the participants answered the Skindex-16 only.

It is important to note that the interval between test and retest should neither be too short, such that respondents might still remember the first answers they gave, nor too long, to allow for significant changes in the assessed condition. Considering that skin symptoms might improve quite quickly after the start of treatment, the interval between the test and retest was set to 3–10 days. Only participants rated stable from the clinical point of view were considered for retesting, i.e., patients without considerable changes in their skin disease as established upon clinical examination.

### Instruments

#### Validation measures

*Dermatology Life Quality Index* (DLQI): this instrument was the first specifically developed to assess HR-QOL in patients with skin diseases [12]. It consists of 10 items distributed across six domains (symptoms and feelings, daily activities, leisure, work and school, personal relationships and treatment) that evaluate the skin disorder over the past week. Respondents are requested to rate how much their skin problem affects different aspects of their lives investigated in the various items. The score of each item varies from 0 (not at all) to 3 (very much); the higher the global score, the poorer the respondent’s QOL. The DLQI has been used to assess QOL in a large number of studies investigating many different skin problems, particularly acne, psoriasis, atopic dermatitis, vitiligo and chronic urticaria [13]. The DLQI was validated for the Brazilian population in 2006 [14].

Hospital Anxiety and Depression Scale (HADS): this instrument was developed by Zigmond and Snaith in 1983 specifically to assess anxiety and depression in patients admitted to non-psychiatric hospitals [15]. It consists of 14 items distributed across two subscales of seven items each, corresponding to depression (HADS-D) and anxiety (HADS-A), respectively. Each item is answered on a four-point Likert scale ranging from 0 (minimum disorder) to 3 (maximum disorder), and the total score of each subscale varies from 0 to 21; higher values indicate greater severity. The HADS has already been validated for the Brazilian population [16].

#### Instrument under validation

Skindex-16: this questionnaire, a single-page, shortened version of Skindex-29, is an easy-to-apply multidimensional instrument consisting of 16 items [10]. Skindex-16 was initially tested with a sample of 500 patients and was shown to be reliable, valid and sensitive to clinical changes. It can be used to assess patients with any skin problem and allows detecting their progression over time after the start of treatment [11]. The items are distributed across three domains—symptoms (items 1 to 4), emotions (items 5 to 11) and functioning (items 12 to 16)–and are answered on a seven-point Likert scale (varying from 0—never bothered, to 6—always bothered), which represents the frequency with which the skin problem bothered the respondent during the past week. The scores are converted to a linear scale ranging from 0 to 100; the higher the scores, the poorer the respondent’s QOL. Access to the questionnaire, or permission to use the Skindex-16, can be found on https://eprovide.mapi-trust.org/instruments/skindex.

### Statistical analysis

#### Clinical feasibility

Clinical feasibility was measured based on the mean (and standard deviation) time needed to the answer the questionnaire and the number of unanswered items. A maximum of 4% of unanswered items was considered acceptable [17]. In order to evaluate the feasibility of the use of Skindex-16 in clinical practice, participants could choose whether to complete the instrument by themselves or applied by the interviewer. Anyway, the interviewer was always available to assist in the process, if needed. The association between the lack of answer to an item (answered vs. unanswered) and a participant’s educational level (low vs. high) was investigated using the chi-square test.

#### Convergent validity

The relationships of Skindex-16 domains with the instruments DLQI, HADS-A and HADS-D were assessed by means of Spearman’s correlation coefficient; values < 0.4, 0.4 to 0.6, and > 0.6 were considered as weak, moderate and strong correlations, respectively [17]. A value of rho > 0.6 was expected for the correlation of Skindex-16 domains (symptoms, emotions and functioning) with the DLQI. In addition, moderate or strong correlations (rho > 0.4) were expected between the Skindex-16 domain emotions and the HADS anxiety and depression subscales. Finally, it was hypothesized that the Skindex-16 domains symptoms and functioning would not be significantly correlated with the HADS subscales (rho < 0.4).

#### Known-groups validity

Based on the hypothesis that the scores on Skindex-16 would be lower among patients with milder skin diseases compared to those with moderate conditions and that the latter’s scores would be lower compared to the patients with severe disorders, the known-groups validity resulted from the comparison of these three groups using the non-parametric Kruskal-Wallis test. The groups were compared using the Mann-Whitney test with Bonferroni correction (considering p-values < 0.017 as significant). Additionally, Skindex-16 scores were compared between patients with inflammatory dermatosis and patients with isolated lesions by means of the Mann-Whitney test.

#### Test-retest reliability

The instrument’s stability was investigated using the intraclass correlation coefficient (ICC), with values over 0.7 considered acceptable [18].

#### Internal consistency

The internal consistency of each Skindex-16 domain was assessed using Cronbach’s alpha; values over 0.70 and under 0.95 were considered acceptable [18].

Further analyses were performed in the subsample of patients with non-neoplastic dermatological diseases; i.e., excluding 13 patients with skin neoplasms (non-melanoma skin cancer, n = 12; melanoma, n = 1).

The statistical analyses were performed in SPSS version 21.0 at a 5% significance level.

### Sample size calculation

*Convergent validity*—calculated based on Pearson’s correlation coefficient, with expected r = 0.6, null hypothesis r = 0.4, error α = 5% and β = 20%; on those grounds, the estimated sample size was 112 participants.

Taking all sample size estimates together, 110 participants were needed, and 29 were to be retested.

## Results

### Sample characteristics

A convenience sampling of 117 participants were invited to participate in the study; 6 refused to participate. The analyzed sample (n = 110) was predominantly composed of females (n = 78, 70.9%), the mean age of the participants was 47.39 years old (standard deviation [SD]: 15.27; min-max: 18.95–87.48), and the largest proportion was married (n = 57, 51.8%). A total of 34 participants (30.9%) had low educational levels, i.e., had not attended school at all or had complete or incomplete primary education only (maximum: 9 years of formal education). For 74.5% of the participants (n = 82), the family monthly income was less than the equivalent of four times the minimum monthly wage in Brazil. The demographic characteristics of the participants are described in Table 1.

**Table 1:** Demographic characteristics of the sample (n = 110).

Characteristic	n (%)
Age (years), mean (SD)	47.39 (15.27)
Gender	
Female	78 (70.9)
Male	32 (29,1)
Site of assessment	
BCH	43 (39.1)
SSF	67 (60.9)
State of residency	
São Paulo	99 (90.0)
Other states	12 (10.0)
Marital status	
Single	32 (29.1)
Married	57 (51.8)
Other	21 (19.1)
Years of formal education	
None	2 (1.8)
1–9	32 (29.1)
9–12	38 (34.5)
> 12	38 (34.5)
Family income*	
< 2	44 (40.0)
≥ 2 < 4	38 (34.5)
≥ 4 < 10	24 (21.9)
≥ 10	4 (3.6)

SD = standard deviation; BCH = Barretos Cancer Hospital;

SSF = São Sebastião Foundation;

* = Brazilian minimum wages (R$).

Main dermatological diagnoses were dermatitis, including atopic, seborrheic, dyshidrotic and contact (n = 15), non-melanoma skin cancer (n = 12), leprosy (n = 11), melasma (n = 11), acne (n = 8), and senile freckle (n = 7). S1 Table details all the dermatological diagnoses. From the 110 patients, there were 23 (20.9%) patients with isolated lesions and 87 (79.1%) patients with inflammatory dermatosis.

### Clinical feasibility

The mean time to answer Skindex-16 was 2 min 41 sec (SD = 51 sec). On the first assessment, the instrument was self-applied for 77 participants (70.0%) and was applied by an interviewer for 33 participants (30%). Item 5 (“*pela persistência/recorrência de sua condição de pele*” [“persistence/recurrence of your skin condition…”]) had a high nonresponse frequency due to doubts or lack of understanding (n = 21; 19.1%). An unplanned analysis with the chi-square test was performed to investigate the association of low educational level with nonresponse to item 5. Of the 21 participants who did not answer item 5, 15 (71.4%) had a low educational level, i.e., no or up to nine years of formal education. Of the 89 participants who answered item 5, 31 (34.8%) had a low educational level (p-value 0.002). No other item posed problems to understanding, and the number of item nonresponse instances was very low. S2 Table shows the descriptive analysis of the scores on Skindex-16 domains, HADS subscales and DLQI in the whole sample.

### Internal consistency

Cronbach’s alpha for Skindex-16 was 0.945 (95% CI = 0.926–0.961). Relative to Skindex-16 domains, the values were 0.867 (95% CI = 0.821–0.904), 0.930 (95% CI = 0.905–0.950) and 0.888 (95% CI = 0.851–0.919) for symptoms, emotions and functioning, respectively. Elimination of items did not significantly influence Cronbach’s alpha. Cronbach’s alpha values were very similar when we analysed separately the internal consistency of Skindex-16 in the subgroups of patients with any skin condition (n = 110) and without skin cancer (n = 97) (S3 Table).

### Test-retest reliability

The ICC values for the Skindex-16 domains symptoms, emotions and functioning were 0.947 (95% CI = 0.875–0.977), 0.860 (95% CI = 0.680–0.936) and 0.843 (95% CI = 0.669–0.926), respectively. Test-retest analyses in subgroups of patients with any skin condition (n = 110) and without skin cancer (n = 97) (S4 Table).

### Convergent validity

The Spearman’s correlation coefficients observed for the relationship between the Skindex-16 domains (symptoms, emotions and functioning) and scores on the HADS-A, the HADS-D and the DLQI are summarized in Table 2. All three Skindex-16 scales exhibited strong correlation with DLQI scores (rho = 0.664, 0.766 and 0.712 for the domains symptoms, emotions and functioning, respectively). Relative to the HADS-A and the HADS-D, the Skindex-16 domain with the highest correlation coefficient was emotions, with moderate correlation (0.4–0.6); the values for the domains symptoms and functioning were low (close to 0.4), as previously hypothesized. Similar findings were observed in separate analysis within subgroups of patients with any skin condition (n = 110) and without skin cancer (n = 97) (S5 Table).

**Table 2:** Convergent analyses between Skindex-16, HADS and DLQI.

Scales of Skindex-16	Instruments	rho (p<0.001)
Symptoms	HADS-A	0.395
	HADS-D	0.395
	DLQI	0.664
Emotions	HADS-A	0.548
	HADS-D	0.555
	DLQI	0.766
Functioning	HADS-A	0.489
	HADS-D	0.456
	DLQI	0.712

HADS-A = Hospital Anxiety and Depression subscale anxiety;

HADS-D = Hospital Anxiety and Depression subscale depression;

DLQI = Dermatology Life Quality Index; rho = Spearman’s correlation coefficients.

### Known-groups validity

As previously hypothesized, the scores differed among the groups classified as having mild, moderate or severe skin diseases and were highest among the latter (Table 3). The Kruskal-Wallis test detected differences among the three groups (emotions: p < 0.001; symptoms: p = 0.002; functioning: p < 0.001). Analysis using the Mann-Whitney test with Bonferroni correction showed differences in all three Skindex-16 domains between the mild and moderate skin disease groups (emotions: p < 0.001; symptoms: p = 0.049; functioning: p < 0.001) and between the mild and severe skin disease groups (emotions: p = 0.002; symptoms: p = 0.001; functioning: p = 0.002). Patients with inflammatory dermatosis presented higher scores on emotions (p = 0.016) and functioning (p = 0.056), but not in symptoms (p = 0.298), when compared with patients with localized lesions (S6 Table).

**Table 3:** Known-groups validation analyses of Skindex-16.

Scales of the Skindex-16 and severity of main skin condition	mean (SD)	median (p25–p75)	p-value*
**Symptoms (4 items)**			0.002
Mild (n = 52)	27.96 (29.29)	20.83 (4.17–41.67)	
Moderate (n = 50)	39.92 (31.09)	35.42 (8.33–62.50)	
Severe (n = 7)	75.60 (26.07)	79.17 (62.50–100.0)	
**Emotions (7 items)**			< 0.001
Mild (n = 52)	40.31 (32.04)	35.71 (12.70–61.90)	
Moderate (n = 50)	70.00 (28.46)	80.48 (54.76–91.67)	
Severe (n = 7)	81.92 (17.95)	77.78 (69.05–100.00)	
**Functioning (5 items)**			< 0.001
Mild (n = 52)	19.10 (26.90)	6.67 (0.00–23.33)	
Moderate (n = 50)	43.77 (28.42)	36.67 (20.00–66.67)	
Severe (n = 7)	61.90 (36.56)	70.00 (23.33–100.00)	

* = Kruskal Wallis test; SD = standard deviation; p25 = percentile 25; p75 = percentile 75.

## Discussion

The present study assessed the psychometric properties of the Portuguese (Brazil) version of Skindex-16. The results indicate that this questionnaire is a valid and reliable instrument to assess the effects of skin disorders on HR-QOL in the Brazilian population.

Many skin problems can have considerable effects on the wellbeing and QOL of patients [19]. Several studies evidenced a relationship between skin diseases and psychiatric disorders, demonstrating that the emotional sphere may be severely affected by several dermatological conditions [20–22]. Skin diseases can have a strong impact on the patient’s social relationships, psychological state and daily activities. The development of valid and reproducible instruments allows for the assessment of several aspects of HR-QOL in the dermatologic setting [23].

The Skindex-16 is a generic dermatology instrument that might be used to evaluate HR-QOL in patients with any skin condition. It is considered an appropriate tool to quantify the burden of dermatosis in order to assist physicians in their clinical practices [11]. The original validation study of the Skindex-16 included 541 patients with several types of dermatosis, such as inflammatory dermatosis, acne, psoriasis, warts and non-melanoma skin cancer. The mean scores of Skindex-16 domains were compared among different dermatosis and the highest scores were reported by patients with psoriasis and other inflammatory dermatosis [10].

The mean time to answer Skindex-16 varied between only two to three minutes. Overall, the instrument was well understood by the respondents, with the only exception being item 5 (“pela persistência/recorrência de sua condição de pele” [“persistence/recurrence of your skin condition…”]), which was not answered by 21% of the sample. The participants were instructed to answer only the items they understood to avoid the “pressure” of having to answer all of the items indiscriminately. Therefore, despite the high frequency of missing responses, item 5 could be considered satisfactory from the psychometric point of view. In this regard, the relevance of cross-cultural adaptation in the translation of instruments for health assessment is emphasized. A panel of experts must assess the equivalence between the translated and original versions, with particular emphasis on the semantic, idiomatic, conceptual and cultural equivalences. Upon considering populations with low educational levels, expert panels often suggest some modifications for translated instruments [4, 24]. The Portuguese (Brazil) version of Skindex-16 used in the present study was translated and provided by the respected Mapi Research Institute, i.e., an organization specializing in the linguistic validation of instruments with results centered on the responses given by patients. Although the translation was perfect from the technical perspective and fully understandable by individuals with high educational levels, participants with lower educational levels, who are common at our institution, had difficulty understanding item 5. Therefore, we suggest that item 5 be modified or even that a new 15-item version of the instrument be considered and tested in future studies in Brazil. [25–30]. None of the mentioned studies reported barriers for understanding item 5 of Skindex-16. However, differently from us, they conducted both translation and cultural adaptation before psychometric evaluation.

Validation is a process that establishes whether an instrument actually measures what it intends to measure. The validation process consists of several steps, and it is expected that convincing evidence will be gathered demonstrating that the instrument in question is useful as a measurement tool (of HR-QOL, for instance) [17]. In the present study, the construct validity of Skindex-16 (Brazilian Portuguese version) was tested based on its convergent and known-groups validity. To assess convergent validity, we investigated the correlation of each Skindex-16 domain (symptoms, emotions and functioning) with the DLQI and HADS anxiety and depression subscales (HADS-A and HADS-D). The values of the correlation coefficients found for the Skindex-16 domains and DLQI were considered clinically significant (over 0.6). The values of the correlation coefficients for the HADS subscales were lower, suggesting moderate correlation with the emotions domain and weak correlation with the symptoms and functioning domains, thus confirming the hypothesis stated *a priori*. Known-groups validity was assessed through the comparison of three clearly different groups (participants with mild, moderate or severe diseases). The scores on all three Skindex-16 domains were lower among the participants with mild disease compared to those with moderate or severe disease, demonstrating that the instrument is adequate to detect clinical differences among different groups. In other validation studies of Skindex-16, the scores of patients with inflammatory skin diseases were higher compared to patients with isolated skin lesions, thus demonstrating poorer QOL in the former [26, 27, 29]. Although our findings are consistent with a poorer QOL in inflammatory dermatosis, the observed differences were only in the emotion and functional domains.

Reliability is a measure of reproducibility; thus, when the patient’s state of health remains stable over time, the results of the assessment instrument should also remain similar [4]. In the present study, the internal reliability of Skindex-16 was assessed based on Cronbach’s alpha, which is a highly relevant method to measure the reliability of scales containing multiple items [17, 18]. The Cronbach’s alpha values were high for all three Skindex-16 domains; elimination of items did not significantly influence these values. The Cronbach’s alpha values found were similar to those obtained in the original validation study, which developed the questionnaire by reducing the number of items in Skindex-29 [10]. Regarding the subsequent validation studies in other languages [25–30], Cronbach’s alpha values were always higher than 0.7, which can be considered acceptable.

Our results relative to test-retest reliability were satisfactory, as the ICC values were above 0.7 for all three Skindex-16 domains. Our findings are in accordance with previous studies [10,30] indicating that Skindex-16 exhibits satisfactory reproducibility.

The present study has some limitations. The first derives from the fact that the study population was recruited at only two centers, both located in the same city. For this reason, it is difficult to generalize the results and state that Skindex-16 is valid for all of Brazil. However, BCH can be considered an excellent setting for the validation of instruments for health assessment in Brazil, as it receives patients from all over the country, especially those with low educational levels. We believe that if an instrument is found to be valid and reliable in this population, it will likely also be useful in populations with higher educational levels. A second limitation derives from the lack of assessment of responsiveness, i.e., the capacity of an instrument to detect clinical changes over time, even when such changes are discrete [18]. Additional studies assessing the responsiveness of Skindex-16 in the Brazilian population are needed, as this capability reflects a relevant psychometric property of instruments intended to be used in studies assessing clinical changes over time (e.g., therapeutic clinical trials).

As study strengths, we highlight the use of a sample composed of patients with skin diseases of various degrees of severity. Thus, Skindex-16 is considered useful for Brazilian patients with any type of skin disease, neoplastic or not. A second strength is the fact that all of the participants were clinically assessed by the investigator, a dermatologist, who established all of the dermatological diagnoses and severity criteria.

## Conclusion

The version of Skindex-16 translated into Brazilian Portuguese is considered valid and reliable for the measurement of the HR-QOL of patients with various types of skin diseases. Skindex-16 can be feasibly used in clinical practice because it is easily understood and quickly answered.

## Supporting information

S1 File Strengthening the Reporting of Observational Studies in Epidemiology (STROBE) statement—Checklist of items. (PDF)

S1 Table Skin conditions of patients participants. (DOC)

S2 Table Descriptive information of Skindex-16, HADS and DLQI. (DOC)

S3 Table Internal consistency values of Skindex-16 domains in the whole sample and in subgroups of patients without skin cancer. (DOCX)

S4 Table Test-retest reliability of Skindex-16 domains in the whole sample and in subgroups of patients without skin cancer. (DOCX)

S5 Table Convergent analyses between Skindex-16, HADS and DLQI in the whole sample and in subgroups of patients without skin cancer. (DOCX)

S6 Table Mean and median scores of Skindex-16 domains according with groups of dermatologic conditions. (DOC)
