# Supplementary material for: The Brazilian version of Skindex-16 is a valid and reliable instrument to assess the health-related quality of life of patients with skin diseases
Source: PLoS One. 2018 Mar 22;13(3):e0194492. doi: 10.1371/journal.pone.0194492 (PMC5864026; doi:10.1371/journal.pone.0194492)
Supplement: S1 File — (PDF) [file pone.0194492.s001.pdf]

***Cheklist STROB*E**

The Brazilian version of Skindex-16 is a valid and reliable instrument to assess the health-related quality of life of patients with skin diseases.

Item no	Recommendation	Pag
1a	**Title and abstract**: Indicate the study's design with a commonly used term in the title or the abstract	1 - 3
1b	**Title and abstract**: Provide in the abstract an informative and balanced summary of what was done and what was found	1 - 3
2	**Background/rationale**: Explain the scientific background and rationale for the investigation being reported	4
3	**Objectives**: State specific objectives, including any prespecified hypotheses	4
4	**Study design**: Present key elements of study design early in the paper	5
5	**Setting**: Describe the setting, locations, and relevant dates, including periods of recruitment, exposure, follow-up, and data collection	5 - 7
6	**Participant**s: *Cross-sectional study*—Give the eligibility criteria, and the sources and methods of selection of participants	5 - 6
7	**Variables**: Clearly define all outcomes, exposures, predictors, potential confounders, and effect modifiers. Give diagnostic criteria, if applicable	6 - 8
8	**Data sources/ measurement**: For each variable of interest, give sources of data and details of methods of assessment (measurement). Describe comparability of assessment methods if there is more than one group	NA
9	**Bias**: Describe any efforts to address potential sources of bias	NA
10	**Study size**: Explain how the study size was arrived at	10
11	**Quantitative variables**: Explain how quantitative variables were handled in the analyses. If applicable, describe which groupings were chosen and why	8 - 10
12 a	**Statistical methods**: Describe all statistical methods, including those used to control for confounding	8 - 10
12 b	**Statistical methods**: Describe any methods used to examine subgroups and interactions	8 - 10
12 c	**Statistical methods**: Explain how missing data were addressed	8 - 10
12 d	**Statistical methods**: *Cross-sectional study*—If applicable, describe analytical methods taking account of sampling strategy	NA
12 e	**Statistical methods**: Describe any sensitivity analyses	NA
13 a	**Results/Participants**: Report numbers of individuals at each stage of study—eg numbers potentially eligible, examined for eligibility, confirmed eligible, included in the study, completing follow-up, and analysed	11
13 b	**Results/ Participants**: Give reasons for non-participation at each stage	NA
13 c	**Results/ Participants** :Consider use of a flow diagram	NA
14 a	**Results/Descriptive data**: Give characteristics of study participants (eg demographic, clinical, social) and information on exposures and potential confounders	10 – 12; Tables 1 and S1
14 b	**Results/ Descriptive data**: Indicate number of participants with missing data for each variable of interest	Table 1 and S1
14 c	**Results/ Descriptive data**: *Cohort study*—Summarise follow-up time (eg, average and total amount)	NA
15	**Results / Outcome data**: *Cross-sectional study*—Report numbers of outcome	NA

	events or summary measures	
16 a	**Results / Main results**: Give unadjusted estimates and, if applicable, confounder-adjusted estimates and their precision (eg, 95% confidence interval). Make clear which confounders were adjusted for and why they were included	12 – 16<br>Tables 2 and 3
16 b	**Results / Main results**: Report category boundaries when continuous variables were categorized	NA
16 c	**Results / Main results**: If relevant, consider translating estimates of relative risk into absolute risk for a meaningful time period	NA
17	**Results / Other analyses**: Report other analyses done—eg analyses of subgroups and interactions, and sensitivity analyses	NA
18	**Discussion / Key results**: Summarise key results with reference to study objectives	16 - 20
19	**Discussion / Limitations**: Discuss limitations of the study, taking into account sources of potential bias or imprecision. Discuss both direction and magnitude of any potential bias	19 - 20
20	**Discussion / Interpretation**: Give a cautious overall interpretation of results considering objectives, limitations, multiplicity of analyses, results from similar studies, and other relevant evidence	18 - 20
21	**Discussion / Generalisability**: Discuss the generalisability (external validity) of the study results	18 - 20
22	**Funding**: Give the source of funding and the role of the funders for the present study and, if applicable, for the original study on which the present article is based	NA
