# Supplementary material for: The Brazilian version of Skindex-16 is a valid and reliable instrument to assess the health-related quality of life of patients with skin diseases
Source: PLoS One. 2018 Mar 22;13(3):e0194492. doi: 10.1371/journal.pone.0194492 (PMC5864026; doi:10.1371/journal.pone.0194492)
Supplement: S1 Table — (DOC) [file pone.0194492.s002.doc]

**Table S1.** **Skin conditions of patients participants.**

| **Dermatological diagnosis** | **n (%)** |
| --- | --- |
| Dermatitis (including atopic, seborrheic, dyshidrotic and contact) | 15 (14.2) |
| Non-melanoma skin cancer | 12 (11.3) |
| Leprosy | 11 (10.4) |
| Melasma | 11 (10.4) |
| Acne | 8 (7.5) |
| Senile freckle | 7 (6.6) |
| Seborrheic keratosis | 4 (3.7) |
| Psoriasis | 3 (2.8) |
| Actinic keratosis | 3 (2.8) |
| Alopecia | 3 (2.8) |
| Folliculitis | 3 (2.8) |
| Rosacea | 2 (1.8) |
| Nail dystrophy | 2 (1.8) |
| Cutaneous drug reactions | 2 (1.8) |
| Vitiligo | 2 (1.8) |
| Skin tags | 1 (0.9) |
| Cutaneous melanoma | 1 (0.9) |
| Dermatitis herpetiformis | 1 (0.9) |
| Pityriasis alba | 1 (0.9) |
| Ephelides | 1 (0.9) |
| Erythema annulare centrifugum | 1 (0.9) |
| Polymorphous light eruption | 1 (0.9) |
| Stretch marks | 1 (0.9) |
| Scabies | 1 (0.9) |
| Hidradenitis suppurativa | 1 (0.9) |
| Neurofibromatosis | 1 (0.9) |
| Subacute cutaneous lupus | 1 (0.9) |
| Prurigo nodularis | 1 (0.9) |
| Onycholysis | 1 (0.9) |
| Melanocytic nevus | 1 (0.9) |
| Porphyria cutanea | 1 (0.9) |
| Viral rash | 1 (0.9) |
| Chronic urticarial | 1 (0.9) |
