# Supplementary material for: The Brazilian version of Skindex-16 is a valid and reliable instrument to assess the health-related quality of life of patients with skin diseases
Source: PLoS One. 2018 Mar 22;13(3):e0194492. doi: 10.1371/journal.pone.0194492 (PMC5864026; doi:10.1371/journal.pone.0194492)
Supplement: S2 Table — (DOC) [file pone.0194492.s003.doc]

**S2 Table.** Descriptive information of Skindex-16, HADS and DLQI (n=110).

| **Instrument / domain** | **Number of items (score range)** | **Mean (SD)** | **Median (p25 - p75)** |
| --- | --- | --- | --- |
|  |  |  |  |
| Skindex-16 symptoms | 4 (0-100) | 36.17 (32.0) | 29.17 (4.17 – 62.50) |
| Skindex-16 emotions | 7 (0-100) | 56.09 (33.79) | 58.33 (23.81 – 85.71) |
| Skindex-16 functioning | 5 (0-100) | 32.86 (31.37) | 21.67 (6.67 – 56.67) |
| HADS-A | 7 (0-21) | 8.57 (5.01) | 8.00 (5.00 – 12.00) |
| HADS-D | 7 (0-21) | 5.96 (4.22) | 5.00 (2.00 – 9.00) |
| DLQI | 10 (0-30) | 6.83 (6.65) | 5.00 (2.00 – 9.50) |

Abbreviations: HADS-A = Hospital Anxiety and Depression Subscale Anxiety; HADS-D = Hospital Anxiety and Depression

Subscale Depression; DLQI = Dermatology Life Quality Index; SD=standard deviation; p25= percentile 25; p75= percentile 75.
