# Supplementary material for: The Brazilian version of Skindex-16 is a valid and reliable instrument to assess the health-related quality of life of patients with skin diseases
Source: PLoS One. 2018 Mar 22;13(3):e0194492. doi: 10.1371/journal.pone.0194492 (PMC5864026; doi:10.1371/journal.pone.0194492)
Supplement: S3 Table — (DOCX) [file pone.0194492.s004.docx]

| **Table S3. Internal consistency values of Skindex-16 domains in the whole sample and in subgroups of patients without skin cancer.** | | |
| --- | --- | --- |
| **Scales of Skindex-16** | **Cronbach’s alpha**  **(CI 95%) whole sample** | **Cronbach’s alpha (CI 95%)**  **sample without skin cancer** |
| Symptoms | 0,867 (0,821 - 0,904) | 0,868 (0,819 - 0,906) |
| Emotions | 0,930 (0,905 - 0,950) | 0,924 (0,896 - 0,947) |
| Functioning | 0,888 (0,851 - 0,919) | 0,881 (0,839 - 0,915) |
|  |  |  |
| CI 95%: confidence interval 95%. | | |
