# Supplementary material for: The Brazilian version of Skindex-16 is a valid and reliable instrument to assess the health-related quality of life of patients with skin diseases
Source: PLoS One. 2018 Mar 22;13(3):e0194492. doi: 10.1371/journal.pone.0194492 (PMC5864026; doi:10.1371/journal.pone.0194492)
Supplement: S4 Table — (DOCX) [file pone.0194492.s005.docx]

| **Table S4. Test-retest reliability of Skindex-16 domains in the whole sample and in subgroups of patients without skin cancer.** | | | | |
| --- | --- | --- | --- | --- |
| **Scales of Skindex-16** | **ICC (IC 95%)**  **whole sample** | | | **ICC (IC 95%) - patients without skin cancer** |
| Symptoms | | 0,947*(0,875-0,977) | | 0,910*(0,725-0,965) |
| Emotions | | | 0,860*(0,680-0,936) | 0,844*(0,337-0,947) |
| Functioning | | | 0,843*(0,669-0,926) | 0,724*(0,486-0,863) |
| ICC= Intraclass Correlation Coefficient; CI 95%= confidence interval 95%; (*) p-value < 0,001. | | | | |
