# Supplementary material for: The Brazilian version of Skindex-16 is a valid and reliable instrument to assess the health-related quality of life of patients with skin diseases
Source: PLoS One. 2018 Mar 22;13(3):e0194492. doi: 10.1371/journal.pone.0194492 (PMC5864026; doi:10.1371/journal.pone.0194492)
Supplement: S5 Table — (DOCX) [file pone.0194492.s006.docx]

| **Table S5. Convergent analyses between Skindex-16, HADS and DLQI in the whole sample and in subgroups of patients without skin cancer.** | | | | |
| --- | --- | --- | --- | --- |
| **Scales of Skindex-16** | | **Instruments** | **rho- whole sample** | **rho- patients without skin cancer** |
| Symptoms | HADS-A | | 0,395* | 0,386* |
|  | HADS-D | | 0,395* | 0,380* |
|  | DLQI | | 0,664* | 0,664* |
| Emotions | HADS-A | | 0,548* | 0,563* |
|  | HADS-D | | 0,555* | 0,541* |
|  | DLQI | | 0,766* | 0,774* |
| Functioning | HADS-A | | 0,489* | 0,512* |
|  | HADS-D | | 0,456* | 0,465* |
|  | DLQI | | 0,712* | 0,701* |
| HADS-A (Hospital Anxiety and Depression Scale-subscale anxiety); HADS-D (Hospital Anxiety and Depression Scale-subscale depression); DLQI (Dermatology Life Quality Index); rho= Spearman’s correlation coefficients; (*) p-value < 0,001. | | | | |
