# Supplementary material for: The Brazilian version of Skindex-16 is a valid and reliable instrument to assess the health-related quality of life of patients with skin diseases
Source: PLoS One. 2018 Mar 22;13(3):e0194492. doi: 10.1371/journal.pone.0194492 (PMC5864026; doi:10.1371/journal.pone.0194492)
Supplement: S6 Table — (DOC) [file pone.0194492.s007.doc]

**S6 Table.** Mean and median scores of Skindex-16 domains according with groups of dermatologic conditions.

| **Skin conditions** | **N** | **Skindex-16**  **symptoms** | | **Skindex-16**  **emotions** | | **Skindex-16**  **functioning** | |
| --- | --- | --- | --- | --- | --- | --- | --- |
|  |  | **Median**  **(p25 – p75)** | **Mean**  **(SD)** | **Median**  **(p25 – p75)** | **Mean**  **(SD)** | **Median**  **(p25 – p75)** | **Mean**  **(SD)** |
| All sample | 110 | 29.2  (4.2 – 62.5) | 36.2  (32.0) | 58.3  (23.8 – 85.7) | 56.1  (33.8) | 21.7  (6.7 – 56.7) | 32.9  (31.4) |
| Isolated lesions^1^ | 23 | 25.0  (4.2-62.5) | 29.5  (29.4) | 38.1  (7.1-80.0) | 41.5  (34.2) | 10.0  (0.0 - 46.7) | 23.7  (28.4) |
| Inflammatory dermatosis^1^ | 87 | 29.2  (8.3-62.5) | 37.9  (32.6) | 64.3  (33.3-90-5) | 59.9  (32.8) | 23.3  (10.0 – 60.0) | 35.3  (31.8) |

^1^ Isolated lesions vs. inflammatory dermatosis: symptoms, p = 0.298; emotions, p = 0.016; functioning, p = 0.056 (Mann-Withney test)
